# Effects of conjugated linoleic acid and high oleic acid safflower oil in the treatment of children with HPV-induced laryngeal papillomatosis: a randomized, double-blinded and crossover preliminary study

**DOI:** 10.1186/1476-511X-11-136

**Published:** 2012-10-12

**Authors:** Louise Louw

**Affiliations:** 1 Department Otorhinolaryngology, Faculty of Health Sciences, University of the Free State, Box339G42, Bloemfontein, 9300, South Africa

**Keywords:** Laryngeal papillomatosis, CLA and HOSF treatments, Disease outcomes, Number of surgical procedures, Scores of the staging system, Immune responses

## Abstract

**Background:**

Surgery is the mainstay therapy for HPV-induced laryngeal papillomatosis (LP) and adjuvant therapies are palliative at best. Research revealed that conjugated-linoleic acid (CLA) may improve the outcome of virally-induced diseases. The effects of Clarinol™ G-80 (CLA) and high oleic safflower oil (HOSF) on children with LP (concomitant with surgery) were evaluated.

**Design:**

A randomized, double-blinded, crossover and reference-oil controlled trial was conducted at a South African medical university. Study components included clinical, HPV type/load and lymphocyte/cytokine analyses, according to routine laboratory methods.

**Participants:**

Overall: ten children enrolled; eight completed the trial; five remained randomized; seven received CLA first; all treatments remained double-blinded.

**Intervention:**

Children (4 to 12 years) received 2.5 ml p/d CLA (8 weeks) and 2.5 ml p/d HOSF (8 weeks) with a washout period (6 weeks) in-between. The one-year trial included a post-treatment period (30 weeks) and afterwards was a one-year follow-up period.

**Main outcome measures:**

Changes in numbers of surgical procedures for improved disease outcome, total/anatomical scores (staging system) for papillomatosis prevention/viral inhibition, and lymphocyte/cytokine counts for immune responses between baselines and each treatment/end of trial were measured.

**Findings:**

After each treatment all the children were in remission (no surgical procedures); after the trial two had recurrence (surgical procedures in post-treatment period); after the follow-up period three had recurrence (several surgical procedures) and five recovered (four had no surgical procedures). Effects of CLA (and HOSF to a lesser extent) were restricted to mildly/moderately aggressive papillomatosis. Children with low total scores (seven/less) and reduced infections (three/less laryngeal sub-sites) recovered after the trial. No harmful effects were observed. The number of surgical procedures during the trial (n6/available records) was significantly lower [(p 0.03) (95% CI 1.1; 0)]. Changes in scores between baselines and CLA treatments (n8) were significantly lower: total scores [(p 0.02) (95% CI −30.00; 0.00)]; anatomical scores [(p 0.008) (95% CI −33.00: -2.00)]. Immune enhancement could not be demonstrated.

**Conclusions:**

These preliminary case and group findings pave the way for further research on the therapeutic potential of adjuvant CLA in the treatment of HPV-induced LP.

## Background

Recurrent laryngeal papillomatosis (RLP) is universally considered a disease with unmet challenges. It is a troublesome and sometimes life-threatening HPV-induced disease (mostly types 6/11), since it may cause airway obstruction in infants and children [1-3]. The true incidence of RLP is unknown, but is estimated between 1 and 4 per 100 000 for USA and Canadian populations. Prevalence is likely variable and depends on age of presentation, socio-economic status of the family, and health care systems of the country or population studied. In South Africa it seems to be prevalent in poor socio-economic communities of rural areas with limited health care facilities [4-7]. The mainstay treatment for this notoriously recurrent disease remains surgery (laser ablation and forceps or microdebrider removal) to retain an open airway. RLP may regress after several surgical procedures or, unpredictably, progress towards highly aggressive papillomatosis (rapid growth) or severe papillomatosis with lung metastasis and dire consequences if left untreated. A tracheotomy is sometimes required until the disease is manageable. Past adjuvant therapies for aggressive growth (dietary supplementations, control of extra-esophageal reflux disease, potent antiviral and chemotherapeutic agents, as well as photodynamic therapies) are palliative at best [8-10]. Proper staging of laryngeal papillomatosis (LP) during micro-laryngoscope examinations is important to follow the clinical course of the disease for treatment options. In children with RLP immunosuppression may influence the clinical course of the disease and hamper current treatments [11,12]. Until an appropriate preventive vaccine is available [13], there is ongoing research for cost effective adjuvant therapies to lift the financial burden this disease places on families and health care systems. In this regard, adjuvant fatty acid therapy may be considered.

There is continuous interest in the effects of conjugated-linoleic acid (CLA) and eicosapentaenoic acid (EPA) on tumor prevention and immune responses, even virally challenged diseases [14,15]. Research revealed that: CLA has anti-proliferative and anti-inflammatory potential; it can induce apoptosis by restoring the PPAR (peroxisome proliferator activator receptor) balance, by up-regulating PPAR-γ and α; and that it can modify mediators of innate and adaptive immune responses, and improve antigen-specific effector functions of both cellular and humoral responses to viral infections [14,16,17]. RLP patients are hallmarked by: enhanced palmitic acid (PA) production, associated with apoptosis and over-expression of PPARδ/β activity [18]; and altered CD8 + counts and a T_H_1/T_H_2 imbalance, associated with development and severity of this HPV-induced disease [19]. Based on the above mentioned information, there is a rationale for CLA to prevent/inhibit papillomatosis or to inhibit/ameliorate HPV infection and, thereby, improve immune defenses and disease outcome of RLP patients [20].

The beneficial use of CLA in human health and diseases is surrounded by controversy and there is ongoing research [21]. In retrospect, CLA may have different effects on human disease conditions, depending on: experimental CLA compositions (different isomers/mixtures) that may have stimulatory or inhibitory effects on cell proliferation and immune responses, as in the case of breast cancer [22]; and ruminant and industrial products of *trans* fatty acids may raise cholesterol levels, as in the case of coronary heart diseases [23]. However, there is ample evidence that specific CLA isomers (*cis*-9, *trans*-11 and *trans*-10, cis-12) have anti-proliferative and immunomodulatory potential, based on *in vitro* and *in vivo* animal and human studies [14,24-27]. CLA (present in dairy products and metabolized in the body) is also commercially available as Clarinol™ G-80 for therapeutic use in clinical trials [14]. A few human clinical trials with CLA on diseases (inflammatory and virally-induced conditions) have seen the light [28,29]. This prompted a preliminary investigation into the therapeutic use of adjuvant CLA (Clarinol™ G-80) as a nutritional supplement after surgery in the case of children with HPV-induced LP.

The main objective of the study was to evaluate the effects of CLA (Clarinol™ G-80) and HOSF (high oleic acid safflower oil) on disease outcomes of LP patients. Aims were to determine whether CLA and HOSF: reduced the number of surgical procedures per year for cost effective treatment; prevented/down-regulated papillomatosis (based on total scores of the staging system); inhibited/ameliorated viral infections (based on anatomical scores of the staging system); and enhanced immune responses (based on T and B lymphocyte and cytokine counts). Preliminary findings of this study (case assessments and group evaluations) indicated therapeutic potential for CLA in the treatment of HPV-induced LP, mainly by: reducing/preventing surgical procedures per year; and by clearing viral infections in mildly/moderately aggressive papillomatosis in children who were not candidates for potential very aggressive growths.

## Methods

### Subjects

The study was approved by the Ethics Committee (Faculty of Health Sciences, University of the Free State). Written informed consent was obtained from the children and their parents/guardians prior to the study. Clear instructions on CLA and HOSF (dosage/time duration/storage) and scheduled visits, were given to caretakers. Dietary handouts (picture guidelines) and proper instructions to follow a basic diet were provided by a nutritionist in an attempt to control the children’s dietary intakes. Patient sampling was hampered by the number of patients that presented during the trial period and the age-restriction included for nutritional purposes. The study period was limited by shelf-values of the products: two years were allowed for optimal quality control. Patients (both genders and aged between 4 to12 years) were recruited from the Free State Province (rural and urban areas). Hospital records of all the children were studied for demographic information and clinical history. Children diagnosed with other diseases (HIV/diabetes/tuberculosis/inflammatory diseases) were excluded. All the children were managed on an out-patient basis, with the exception of one child (in-ward patient).

### Study design

**Figure 1:**
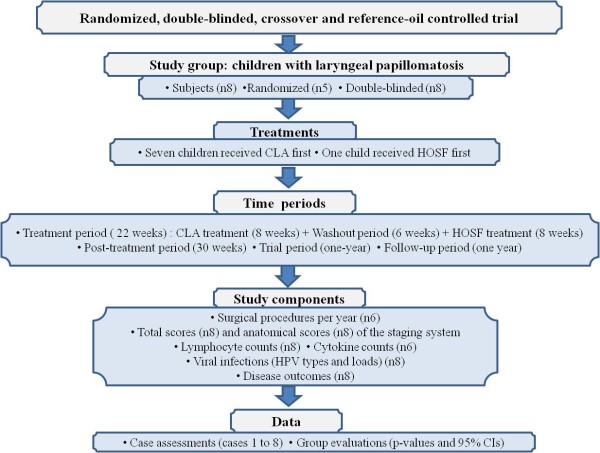
Flow diagram for study design.

A randomized, double-blinded, crossover and reference-oil controlled trial was designed. Products (Clarinol™ G-80 and HOSF) were bottled by a pharmacist (Paraxell, University of the Free State) and the computer developed randomization list was kept confidentially (patients/caretakers/assessors were blinded). Children had to take 2.5 ml p/d of product A (or B) for 8 weeks (Period 1) and 2.5 ml p/d of product B (or A) for 8 weeks (Period 2), with a washout period of 6 weeks in-between. The one-year trial period consisted of CLA and HOSF treatments with a washout period in-between (22 weeks), and a post-treatment period (30 weeks). Ten children enrolled, but eight children completed the trial (two children did not return after commencement of the trial). Among the eight children, two patients were newly recruited with no previous surgical records available. Five children remained randomized, because three children started with period 2. The end result was that all the patients received CLA first in the crossover study (with the exception of one child), as revealed by the statistician. Consequently, the baselines with which all the children started were used for statistical analyses. See flow diagram of the study design depicted in Figure 1.

### Therapeutic products

Clarinol™ G-80 is oil high in *cis*-9, *trans*-11 and *trans*-10, *cis*-12 CLA isomers (50:50 ratio), mainly in triglyceride form. Clarinol™ G-80 is made from natural safflower oil by a gentle, proprietary process and contains concentrated CLA, rosemary extract and vitamin E. High quality CLA when consumed at 3–6 g/d is safe for human consumption [30]. A dosage of 2.5 ml/d equals 2.5 g/d. HOSF is often used as a reference-oil for lipid studies and clinical trials [31,32]. CLA is known for its ability to induce apoptosis and to enhance immune responses [14]. HOSF is known for its nutritional benefits and its oleic acid component can improve antioxidant conditions. Although olive oil can prevent cell proliferation or boost the immune system, disparate findings with oleic acid (main component of olive oil) were encountered in the literature [33,34]. Decision-making for dosage and time durations (both CLA and HOSF treatments) to conduct this trial was within boundaries for previous human trials.

### Surgical procedures and disease staging

Before commencement of the trial all the children were endoscopically diagnosed, staged for disease assessment, and received surgical procedures. All the children were subjected to micro-laryngoscopic examinations during the trial (before and after the first treatment/before and after the second treatment/during washout and post-treatment periods) for clearance of airway passages, if required. All surgical procedures (before trial/during each treatment/after trial/after follow-up period) were carefully documented. Disease assessment was done according to clinical, anatomical and total scores of the Derkay staging system. The total score (i.e. summation of clinical and anatomical scores) gives a fair account of the overall disease condition; the clinical score reflects disease symptoms; and the anatomical score relates to the extent of viral infection. Score sheets for disease staging: before and after each treatment; after the trial; and upon emergency during the trial, were completed and filed. Patients were scheduled for visits to the otorhinolaryngology clinic on a monthly basis during treatment periods to monitor medicine intakes, general well-being of the children, and status quo of the disease. Otherwise, children were seen on emergency.

### Biopsies and blood specimen

Biopsies of the papillomata (size varied, according to availability) were taken endoscopically (before and after the trial) for HPV typing and viral load determinations. Blood specimens were collected for T lymphocyte, B lymphocyte and cytokine analyses (before and after each treatment, as well as after the trial). All specimens were taken according to strict protocol and immediately transferred for routine laboratory analyses. The study components are presented in Figure 1.

### Determination of HPV types and viral loads

The HPV type and viral load of each patient were required for correlation with aggressiveness and disease outcome. The techniques followed for HPV typing were performed according to a method described in the literature [35]. Papilloma biopsies were immediately processed or frozen at −20°C, until analyzed. Briefly, total DNA was extracted from biopsies using QIAamp DNA Mini Kit, according to the manufacturer’s instructions (QIAGEN Inc., Valencia, CA). Consensus primers, previously identified for a region of the genome that is well conserved for most HPV types, were used to amplify a region of the major viral capsid L1 gene, using a standard PCR technique. The primer pair (designated MY09 and MY11) target and amplify a 469 base pair (bp) region between positions 6722 to 7190 of the HPV-6 genome. The nucleotide sequence of amplicons was determined using Big Dye™ Terminator Sequencing Ready Reaction kits with AmpliTaq DNA polymerase FS (Applied Biosystems, Foster City, CA), according to the manufacturer’s instructions. Nucleotide sequence data was edited using ChromasPro Version 1.42 and aligned using Clustal Version X1.8. The genotypes of HPV were determined by BLAST analysis. HPV viral loads were determined on baseline samples and samples submitted after the one year trial period. Quantification of HPV viral loads was determined by use of a relative quantitative real time PCR based on SYBR Green technology. The viral load was determined using the primers mentioned and a modification of methods described in the literature [36,37], in which amplification of the B globin gene was used as an external control and the viral load expressed as a ratio of HPV to total DNA.

### Lymphocyte assay

Peripheral blood specimens were collected in purple stopper tubes (sterile K3 EDTA vacutainers) and analyzed within 24 h by flow cytometry. Mature human T-lymphocytes (CD3+), suppressor/cytotoxic T-lymphocyte subsets (CD3 + CD8+), and helper/inducer T-lymphocytes subsets (CD3 + CD4+) were identified and determined in erythrocyte-lysed whole blood. T lymphocyte counts were determined by a BD FACS Caliber Flow Cytometer and the use of MultiTEST™ reagents and TruCOUNT tubes, according to manufacturer’s instructions Results were reported as the percentage of positive cells per lymphocyte population or as the number of positive cells/μl of blood (absolute count) by using MultiSET™ software.

### Immunoglobulin assay

Peripheral blood specimens were collected in yellow stopper tubes (BD Vacutainer® SST™ II Advance) without stasis, and centrifuged within 2 h to separate serum from cells for immediate immunoglobulin (Ig) analyzes. Techniques followed according to manufacturer’s instructions were: IgG, IgA and IgM were analyzed on the Dade Behring BN Prospect Nephelometer by using an immunonephelometric technique with reagents from Dade Behring (N Antisera to Human immunoglobulins G, A and M); and IgE was analyzed using a particle-enhanced immunonephelometric technique with reagents from Dade Behring (N Latex IgE mono). Results were evaluated by comparison with a standard of known concentration and an internal quality control procedure was performed to ensure optimal results.

### Cytokine assay

Peripheral blood specimens were collected in green stopper tubes (vacutainers containing heparin as anti-coagulant) for cell culturing. Peripheral blood mononuclear cells (PBMCs) were cultured in a medium (containing L-glutamine, 20% fetal calf serum and 0,004 mg/ml gentamycin) stimulated with phytohaemagglutinin M (PHA-M) and then centrifuged to obtain the supernatant for cytokine analyses, according to methods described in the literature [31,32]. The cytokine assay was performed by flow cytometry with the use of cytometric bead arrays (Becton Dickinson), according to manufacturer’s instructions. A cytometric bead array kit (Human Th1/Th2 Cytokine Kit II, BD Biosciences, San Jose, California) was used, and results were analyzed with BD FCAP Array™ software (Cat.No.641488). Concentrations of interleukin (IL-2, IL-4, IL-6, IL-10), tumor necrosis factor-α (TNF-α), and interferon-γ (IFN-γ) levels were measured. The limit of detection for each cytokine was defined as the corresponding concentration at two standard deviations above the median fluorescence of 20 replicates of the negative control (0 pg/ml).

### Statistical analyses

For statistical comparisons the Mann–Whitney or paired non-parametric signed rank tests were used where applicable. Results were summarized by medians, quartiles, minima and maxima and the differences between medians (p-values) and 95% confidence intervals (95% CI) were calculated.

## Results and discussions

### General

Demographic information regarding gender, age, disease duration, and residential area of the patients (cases 1 to 8) is presented in Table 1. Children were diagnosed upon hospital admittance and onset of the disease (age of the child at the time) was not always assessable for correlation with disease outcome. All the children underwent micro-laryngoscopic surgery with cold steel debulking and/or CO2 laser removal, with the exception of one child (Case 1) where a microdibrider was also used. Only one child (Case 1) had a tracheotomy several years prior to the trial. None of the patients previously received any adjuvant therapies, with the exception of one child (Case 1) who had zinc supplementation three years prior to the trial. Disease outcomes were assessed: firstly, as cases (1 to 8); and secondly, as groups (where applicable for different components of the study).

**Table 1:** Demographic information of laryngeal papillomatosis patients

**Patients**	**Gender**	**Age**		**Disease duration**	**Residential area**
		**Diagnosis**	**Trial**		
Case 1	Male	4 yrs	11 yrs	± 7 yrs	Rural
Case 2	Male	6 yrs	8 yrs	± 2 years	Rural
Case 3	Male	6 yrs	11 yrs	± 5 years	Urban
Case 4	Female	8 yrs	9 yrs	± 1 year	Urban
Case 5	Male	9 yrs	9 yrs	New patient	Rural
Case 6	Male	7 yrs	7 yrs	New patient	Rural
Case 7	Female	1 yr	4 yrs	±3 years	Urban
Case 8	Male	3 yrs	11 yrs	±8 yrs	Rural

### Number of surgical procedures

#### Case assessments

During each treatment (CLA and HOSF) none of the children required surgical procedures. After the one-year trial period two children (cases 1 and 2) (25%) had surgical procedures during the post-treatment period, while six children (cases 3/4/5/6/7/8) (75%) had no surgical procedures. After the one-year follow-up period three children (cases 1/2/6) (37.5%) had recurrence (several surgical procedures) and five children (cases 3/4/5/7/8) (62.5%) recovered. During the one-year follow-up period four of the five children (cases 3/5/7/8) had no surgical procedures, but one child (case 4) required one more surgical procedure (one month after the trial) before recovery. All the children received surgical procedures before each treatment, with the exception of cases 3 and 6 who were clear before HOSF treatment. The numbers of surgical procedures (before trial/after each treatment/after trial/after follow-up period) are indicated in Table 2.

**Table 2:** Numbers of surgical procedures and disease outcomes

**Patient**	**Before trial**	**During CLA**	**During HOSF**	**During trial**	**During follow-up**	**Disease outcomes**
Case 1·	7.4	0	0	5	10	Recurrence
Case 2·	10.2	0	0	2	12	Recurrence
Case 3	2	0	0	0	0	Recovered
Case 4◦	4.6	0	0	0	1	Recovered
Case 5*	0	0	0	0	0	Recovered
Case 6*	0	0	0	0	6	Recurrence
Case 7	2.7	0	0	0	0	Recovered
Case 8	1.1	0	0	0	0	Recovered

· Cases 1 and 2 received surgical procedures during post-treatment period of one-year trial; ◦ Case 4 recovered one month after trial; * Cases 5 and 6 were new patients with no available surgical records.

The washout period between CLA and HOSF treatments was considered sufficient [31]. No adverse effects were observed with the sequence of treatments (case 8 received HOSF first), despite a previous *in vitro* study that indicated interference when CLA was supplemented after oleic acid [38]. Case 1 (severely aggressive with lung metastasis) and case 2 (highly aggressive with rapid recurrence) had lower or a remarkable lower number of surgical procedures during the trial (post-treatment period), compared with those before the trial (average per year). Case 4 who recovered during the follow-up period was considered more aggressive before the trial, based on the average number of surgical procedures per year (> 4 per year) this child received; Case 6 (a newly recruited patient) had rapid recurrence after the trial and received 6 surgical procedures during the one-year follow-up period (see Table 2). The outcome of case 6 correlated with its viral status (see viral infections).

#### Group evaluation

Among the study group two patients were newly recruited with no surgical records available. For those patients with surgical records (n6) the number of surgical procedures during the one-year trial, compared with those before the trial over a period of 13–105 months (calculated from dates of diagnoses), was significantly lower (p 0.03) (95% CI 1.1; 0) (Table 3). This finding may be in favor of the beneficial use of CLA to reduce the number of surgical procedures for cost effective treatment of this notoriously recurrent disease.

**Table 3:** Surgical procedure values before and after the trial (n6)

**Period**	**MED**	**LQ**	**UQ**	**MIN**	**MAX**
Before trial	3.7	2	7.4	1.1	10.2
After trial	0	0	1	0	5

MAX: maximum MED: median; MIN: Minimum; Log diff: log difference LQ: lower quartile; UQ: upper quartile.

Significant reduction in surgical procedures during trial (12 month period) (p 0.03, 95% CI 1.1; 0), compared with average surgical procedures per year before the trial (from diagnosis: 13–105 month period).

### Viral infections

Children were typed as HPV6 or 11. Overall: cases typed HPV6/11 with lower viral loads and no surgical procedures during the trial recovered; while those typed HPV6/11 with higher viral loads and/or no surgical procedures during the trial had recurrence. HPV11 infection is considered to confer more aggressive growth. Of significance may be the fact that case 7 (HPV11/low viral load before the trial) recovered during the trial, while case 6 (HPV11/highest viral load before the trial) had recurrence after the trial. Case 4 (HPV6) who recovered during the one-year follow-up period (20 weeks later/one more laser treatment) had a high viral load after the one-year trial, while case 2 (HPV6) (marked by rapid recurrence before and after the trial) had the highest viral load after the trial. HPV typing and viral loading (before and after the trial) for each patient is summarized in Table 4. Viral loads before and after the trial were not significantly different (p 0.95) (95% CI8 -0.80; 2.31) (Table 5). The role of viral loading rather than type for disease aggressiveness needs further clarification.

**Table 4:** HPV types and viral loads of laryngeal papilloma biopsies

**Patients**	**HPV type**	**Viral load before trial**		**Viral load after trial**	
		**HPV: DNA**	**Log-value**	**HPV: DNA**	**Log-value**
Case 1	HPV11	76.14	1.88	11.92	1.08
Case 2	HPV6	1620	3.21	227000	5.35
Case 3	HPV6	369	2.57	690	2.84
Case 4	HPV6	229	2.36	46500	4.67
Case 5	HPV6	1900	3.28	565	2.75
Case 6	HPV11	59900	4.78	11700	4.07
Case 7	HPV11	1.18	0.55	Clear	Clear
Case 8	HPV6	1.23	0.0899	Clear	Clear

**Table 5:** Viral load log values before and after the trial (n8)

**Period**	**MED**	**LQ**	**UQ**	**MIN**	**Max**
Baseline	2.47	1.21	3.25	0.09	4.78
End trial	2.8	0.54	4.37	0.00	5.35
Log diff	−0.31	−0.63	1.21	−0.80	2.31

MAX: maximum MED: median; MIN: Minimum; Log diff: log difference LQ: lower quartile; UQ: upper quartile.

### Scores of the staging system

#### Case assessments: total scores

Total scores (after each treatment/after the trial) give an overall account of how papillomatosis was prevented or down-regulated in each child (Table 6). Overall: papillomatosis was prevented in cases 3/4/5/6/8 after CLA treatment; cases 3/4/7 after HOSF treatments; and Case 7 after the trial. After CLA treatment a decrease in total scores occurred among all the children, except case 2 (same score); after HOSF treatment total scores varied, but there appeared to be a slight decrease in patients with lower total scores (except case 6). This patient was considered potentially more aggressive before the trial (HPV type 11/highest viral load among the group) (see Table 4). CLA treatment prevented papillomatosis effectively in those patients who received CLA first (cases 3/4/5/6) and the patient who received HOSF first (case 8) in the crossover study. Children (cases 3/5/7/8) with total scores of seven and less recovered after the trial (without further treatment), except case 4 (see Table 6). This patient was considered more aggressive before the trial (> 4 surgical procedures per year) (see Table 2).

**Table 6:** Laryngeal papillomatosis: scores of the staging system

**Clinical scores**								
	Case 1	Case 2	Case 3	Case 4	Case 5	Case 6	Case 7	Case 8
Before CLA	3	2	1	1	1	0	4	1
After CLA	6	4	0	0	0	0	1	0
Before HOSF	9	10	0	1	1	0	0	1
After HOSF	5	1	0	0	1	2	0	0
After trial	5	4	1	0	2	4	0	4
**Anatomical scores**								
	Case 1	Case 2	Case 3	Case 4	Case 5	Case 6	Case 7	Case 8
Before CLA	43	28	12	12	3	5	10	7
After CLA	10	26	0	0	0	0	2	2
Before HOSF	39	12	0	12	8	0	2	7
After HOSF	47	15	0	0	1	12	0	2
After trial	48	18	6	4	4	33	0	3
**Total scores**								
	Case 1	Case 2	Case 3	Case 4	Case 5	Case 6	Case 7	Case 8
Before CLA	46	30	13	13	4	5	14	8
After CLA	16	30	0	0	0	0	3	0
Before HOSF	48	22	0	13	9	0	2	8
After HOSF	52	16	0	0	2	14	0	3
After trial	53	22	7	4	6	37	0	7

All the children received CLA first (except case 8) in the double-blinded, crossover study.

#### Case assessments: anatomical scores

Anatomical scores after each treatment give an account of how viral infections were cleared or inhibited (Table 6). A summary of the anatomical sites and sub-sites provide insight into the areas of infection before and after the trial (Table 7). Overall: viral infections were cleared in cases 3/4/5/6 after CLA treatment; cases 3/6 after HOSF treatment; and case 7 after the trial (Table 6). Patients with primarily infection of the larynx (3 and less sub-sites) recovered after the trial (cases 3/4/5/8 typed HPV6) (Table 7). Main trends observed were: case 7 (HPV11/lowest viral load before the trial) recovered sooner (after both treatments); case 4 (HPV6/highest viral load after the trial) was clear after both treatments, presented with a low score after the trial, but recovered early during the follow-up period. The disease condition of case 1 (multi-site infections) and that of case 2 (multiple laryngeal sub-site infections) remained approximately the same as before the trial (Table 7). The prognosis of case 1 with lung metastasis is poor. The disease condition of case 2 can be ascribed to ill-health (malnutrition) and poor parental care, as witnessed during scheduled visits to the clinic. The immunocompetence of cases 1 and 2 were questionable.

**Table 7:** Summary of anatomical site and sub-site infections before and after the trial

Case 1	Before: Larynx (aryepiglottis fold/false and true vocal cords/arytenoids/anterior and posterior commissures/sub-glottis) and trachea/lungs
	After: Larynx (aryepiglottis folds/false and true vocal cords/anterior and posterior commisure/sub-glottis) and trachea/bronchi/lungs
Case 2	Before: Larynx (aryepiglottis folds/false and true vocal cords/arytenoid/anterior commissure/sub-glottis)
	After: Larynx (aryepiglottis fold/false vocal cords/true vocal cord)
Case 3	Before: Larynx (true vocal cords/anterior and posterior commissures)
	After: Larynx (true vocal cord/anterior commissure)
Case 4	Before: Larynx (false and true vocal cords/arytenoid), palate and pharynx
	After: Larynx (true vocal cords)
Case 5	Before: Larynx (true vocal cords/anterior commissure)
	After: Larynx (false vocal cords/true vocal cord)
Case 6	Before: Larynx (aryepiglottis fold/arytenoid/posterior commissure) and pharynx
	After: Larynx (laryngeal epiglottis surface/aryepiglottis folds/false and true vocal cords/arytenoids/anterior and posterior commissures)
Case 7	Before: Larynx (false vocal cord/true vocal cord/anterior and posterior commissures)
	After: Clear
Case 8	Before: Larynx (laryngeal epiglottis surface/aryepiglottis fold/arytenoid/anterior commissure)
	After: Larynx (anterior and posterior commissures)

In retrospect, the effects of CLA (and HOSF in this crossover study) on total and anatomical scores were restricted to cases with low total scores. Research revealed that olive oil (rich in oleic acid) has potential to inhibit CO_2_ activity and Bcl oncogene-expression during early tumorigenesis, but this could not be confirmed for pure oleic acid [39]. Taken in consideration the benefits of oleic acid and safflower oil in HOSF, it is possible that both CLA and HOSF may have contributed to disease outcomes during this crossover trial, depending on disease aggressiveness. Future research with CLA and HOSF requires parallel investigations for elucidation of their individual effects on LP patients.

#### Group evaluations: total and anatomical scores

Changes in total scores between baselines and CLA treatment were significantly lower (p < 0.02) (95% CI −30.00; 0.00), but not significantly different between baselines and HOSF treatment (p 0.47) (95% CI −13.00; 14.00). Differences in changes between treatments were also not significantly different (p 0.13) (95% CI −34.00; 6.00). Changes in anatomical scores were significantly lower between baselines and CLA treatment (p 0.008) (95% CI −33.00; -2.00), but not significantly different between baselines and HOSF treatment (p 0.95) (95% CI −12.00; 12.00). Differences in changes between treatments were significantly higher (p 0.05) (95% CI −41.00; 4.00). Changes between baselines and the end of the trial for total and anatomical scores were not significantly different. Group values for total and anatomical scores are presented in Tables 8 and 9.

**Table 8:** Total score values of the staging system (n8)

**Baseline scores**				
MED	LQ	UQ	MIN	MAX
13.00	6.50	22.00	4.00	46.00
**Changes in scores after CLA treatment**				
Med	LQ	UQ	Min	Max
0.00	0.00	9.50	0.00	30.00
**Changes in scores between baseline and CLA treatment**				
MED	LQ	UQ	MIN	MAX
−9.50	−13.00	−4.50	−30.00	0.00
**Changes in scores after HOSF treatment**				
Med	LQ	UQ	Min	Max
2.50	0.00	15.00	0.00	52.00
**Changes in scores between baseline and HOSF treatment**				
Med	LQ	UQ	Min	Max
−3.50	−6.50	2.00	−13.00	14.00
**Differences in changes between treatments**				
Med	LQ	UQ	Min	Max
−6.00	−16.00	1.50	−34.00	6.00

MAX: maximum MED: median; MIN: Minimum; LQ: lower quartile; UQ: upper quartile.

**Table 9:** Anatomical score values of the staging system (n8)

**Baseline scores**				
MED	LQ	UQ	MIN	MAX
11.00	6.00	20.00	3.00	43.00
**Changes in scores after CLA treatment**				
Med	LQ	UQ	Min	Max
0.00	0.00	6.00	0.00	26.00
**Changes in scores between baseline and CLA treatment**				
MED	LQ	UQ	MIN	MAX
−7.50	−12.00	−4.00	−33.00	−2.00
**Changes in scores after HOSF treatment**				
Med	LQ	UQ	Min	Max
1.50	0.00	13.50	0.00	47.00
**Changes in scores between baseline and HOSF treatment**				
Med	LQ	UQ	Min	Max
−1.00	−6.00	5.50	−12.00	12.00
**Differences in changes between treatments**				
Med	LQ	UQ	Min	Max
−5.50	−14.50	−1.00	−41.00	4.00

MAX: maximum MED: median; MIN: Minimum; LQ: lower quartile; UQ: upper quartile.

The significant lower total scores after CLA treatment indicate CLA potential to prevent/down-regulate papillomatosis during treatment. The significant lower anatomical scores after CLA treatment indicate CLA potential to inhibit/ameliorate viral infections during treatment. The significant higher anatomical scores between treatments (CLA and HOSF) also indicate that CLA treatment was effective in inhibiting viral infections during treatment, but not afterwards. The question that can be raised is did CLA contribute to disease remission/recovery or was it spontaneous remission/recovery? There is ample evidence in the literature that CLA can prevent colon polyps (growths) after surgery [16,24,40]. It was also reported that CLA can significantly improve the conditions of rheumatoid arthritis patients (same dosage/same CLA mixture of isomers) [28]. Pre-clinical and clinical trials also confirmed the beneficial use of CLA in the treatment of virally-induced asthma [41]. It is feasible that CLA therapy (after surgery) may be useful in the treatment of HPV-induced LP patients, but more extensive research that includes distinct categories for aggressiveness concomitant with full surgical history is required for proper assessments.

### Disease outcomes and aggressiveness

There is a bevy of criteria for disease aggressiveness, namely: HPV type 11 and/or high viral load; frequent surgical procedures (> 4 surgical procedures per year); disease extent (multiple site/sub-site infections); rapid recurrence (repeated surgical procedures); early age of onset; and prolonged disease duration [1-3]. The patients of this study had either mildly/moderately or highly/severely aggressive papillomatosis, according to lower or higher total scores (see Table 6).

Disease outcomes of the patients (cases 1 to 8) based on criteria for aggressiveness before and after the trial are summarized:

Case 1 (HPV11): severely aggressive before the trial (HPV type/high total score/repeated surgical procedures); recurrence during post-treatment period, after the trial and during the follow-up period (multi-site and sub-site infections/repeated surgical procedures).

Case 2 (HPV6): highly aggressive before the trial (high total score/rapid recurrence/repeated surgical procedures); recurrence during post-treatment and follow-up periods (higher viral load after trial/repeated surgical procedures).

Case 3 (HPV6): moderately aggressive before the trial (low viral load/moderate total score/< 4 surgical procedures per year); recovered after the trial (low viral load/low total score/no surgical procedures).

Case 4 (HPV6): potentially more aggressive before the trial (>4 surgical procedures per year/moderate total score); recurrence after the trial (higher viral load/low total score/no surgical procedures), but recovered after one more surgical procedure (one month later during follow-up period).

Case 5 (HPV6): Mildly aggressive before the trial (low total score); recovered after the trial (lower viral load/low total score/no surgical procedures).

Case 6 (HPV11): potentially more aggressive before the trial (HPV type/high viral load); highly aggressive after the trial (high viral load/high total score/rapid recurrence/repeated surgical procedures during follow-up period).

Case 7 (HPV11): potentially more aggressive before the trial (HPV type/moderate total score); recovered after the treatment periods (low viral load/no surgical procedures).

Case 8 (HPV6): mildly aggressive before the trial (low viral load/low total score/< 4 surgical procedures per year); recovered after the trial (low viral load/low total score/no surgical procedures).

### Immune responses

#### Case assessments

**Figure 2:**
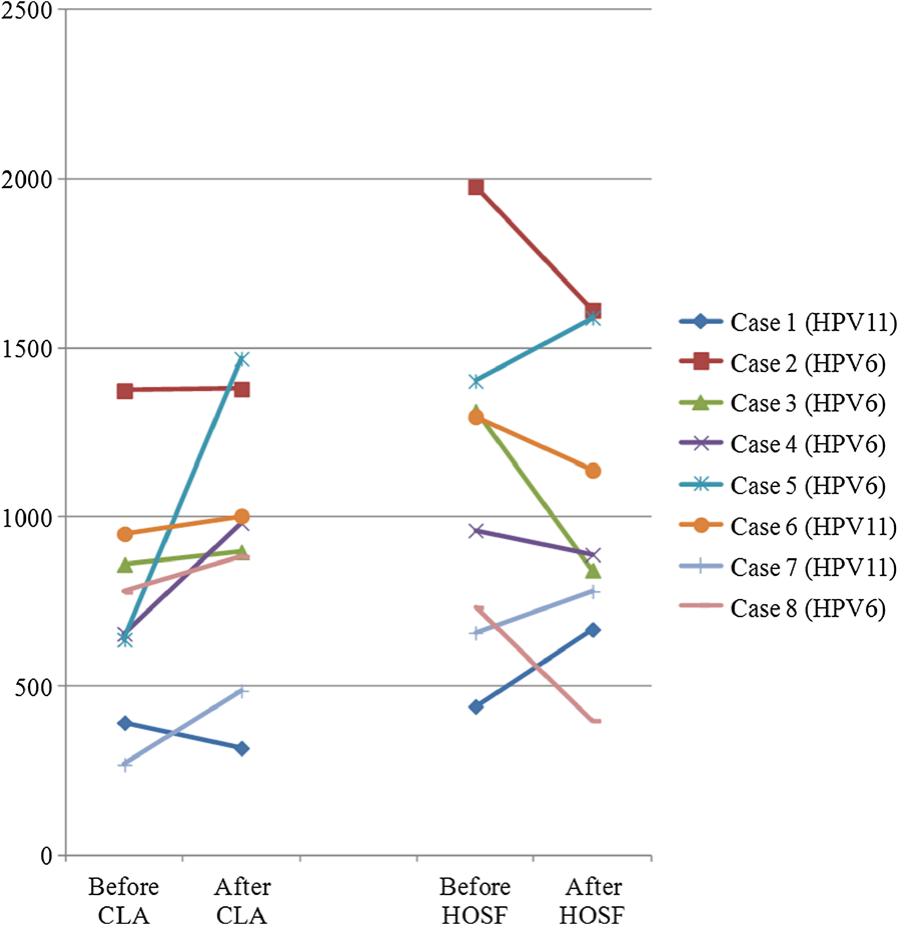
CD8^+^ counts before and after treatments.

**Figure 3:**
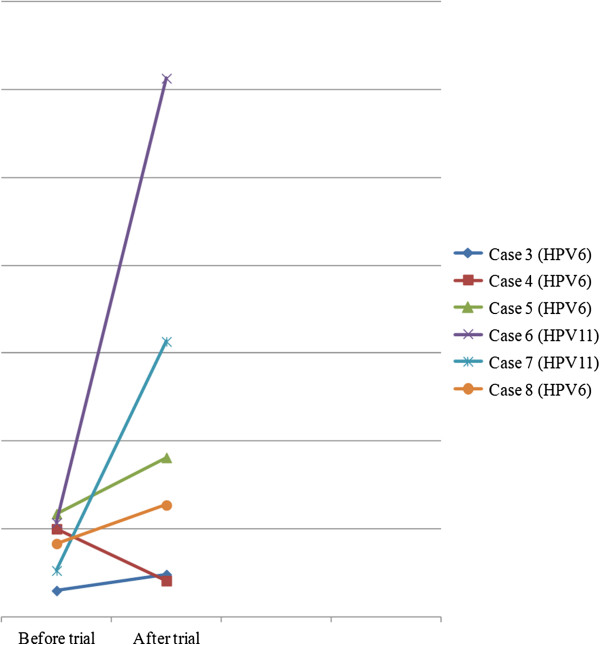
**IL-10 counts before and after the trial.** Case 6 (HPV11/highest viral load before trial) had highest IL-10 count after trial; Case 4 (HPV11/very low viral load before trial) had lowest IL-10 count after trial.

Upon studying the lymphocyte and cytokine profiles of the cases (after each treatment/after the trial) it was clear that: counts varied little; were within available reference ranges; were mostly inconsistent; and mostly no correlations with disease aggressiveness occurred (data not shown) (Additional files 1, 2 and 3). This is to be expected, because of the few cases studied. Another study with CLA supplementation on human rhinovirus (HRV) infection also showed inconsistent results, mainly because of insufficient patient numbers for correlations with illness symptoms within the group [29]. In the literature it is reported that CLA supplementation can increase CD8^+^/IL-2/IL-10 counts in humans [14]. Previous studies on LP patients reported on the significance of CD8^+^/IL-2/IL-10 in the clinical course of this disease [19,42,43]. Therefore, the current study focused specifically on changes in CD8^+^IL-2/IL-10 counts after treatments. Main trends that occurred among the LP cases are briefly mentioned. An increase in CD8^+^ counts was observed after CLA treatment in some patients (cases 3 to 8) (Figure 2). The increase in CD8^+^ counts may reflect an improved immune response for these children that lasted beyond the period of treatment, but were short-lived. It was established that the effects of CLA-60 supplemented diet (compared with a soybean diet) on immune cell phenotype (i.e. numbers of CD8^+^ counts) lasted approximately 2 months after the product was withdrawn, but effector functions (i.e. antigen-stimulated proliferation and cytotoxicity) disappeared earlier (approximately 3 weeks), based on an animal model study where pigs were inoculated with a virus [44]. With respect to IL-10 counts, it can be mentioned that: case 4 (HPV6/high viral load after trial/low total score after trial) presented with a lower IL-10 count after the trial, but recovered during the follow-up period; while case 6 (HPV11/highest viral load before the trial/high total score after the trial) presented with a very high IL-10 count after the trial, but had recurrence during the follow-up period (Figure 3). However, all the other children (cases 3/5/7/8) with higher IL-10 counts after the trial (study component excluded cases 1 and 2) recovered (Figure 3). It is feasible that enhanced IL-10 may successfully combat viral infections in mildly/moderately aggressive papillomatosis, but not in potentially very aggressive papillomatosis marked by higher viral loading.

#### Group evaluations

Comparisons of CD8^+^/IL-2/IL-10 counts, namely: changes in counts between baselines and each treatment; the difference in changes between treatments; and changes between baselines and the end of the trial, showed that none of the counts were significantly different, with one exception. Significantly higher IL-10 counts (p 0.03) (95% CI 66.33; 1011.57) (n6 patients) occurred after the one-year trial (Table 10). Of significance is that RLP patients are characterized by high IL-10 levels [42]. It is also known that enhanced IL-10 has the potential to inhibit other cytokines (IL-2/IL-4/IL-6/IL-8 and TNF-α) [45].

**Table 10:** CD8 + and interleukin-2 and −10 count values (n6)

**Baseline counts**					
Subsets	MED	LQ	UQ	MIN	MAX
CD 8	715	513.5	904.5	266	1373
IL-2	7.18	5.85	8.5	4.51	9.14
IL-10	136.22	30.04	214.39	0	234.86
**Difference in counts after CLA treatment**					
Subsets	MED	LQ	UQ	MIN	MAX
CD 8	45	−35.5	275	−334	832
IL-2	0.73	−1.16	1.72	−3.21	5.21
IL-10	4.31	−76.74	62.57	−211.63	181.03
**Difference in counts after HOSF treatment**					
Subsets	MED	LQ	UQ	MIN	MAX
CD 8	16.5	−261.5	155.5	−470	227
IL-2	−0.74	−7.16	1.01	−10.36	5.21
IL-10	54.27	−77.79	352.93	−125.04	615.78
**Difference in counts between CLA and HOSF treatments**					
Subsets	MED	LQ	UQ	MIN	MAX
CD 8	290.5	−103.5	454	−439	644
IL-2	1.85	−2.86	6	−4.22	15.57
IL-10	−46.05	−564.56	138.28	−579.81	187.61
**Difference in counts between baselines and end of the trial**					
Subsets	MED	LQ	UQ	MIN	MAX
CD 8	270	209.5	395	−118	1327
IL-2	−2.45	−5.85	20.07	−7.01	144.39
IL-10	107.82	82.39	521.35	66.33	1011.57

MAX: maximum MED: median; MIN: Minimum; LQ: lower quartile; UQ: upper quartile.

Upon studying the literature, studies with CLA supplementation on healthy individuals without or with vaccinations were encountered. The effects of CLA supplementation on immune function in young healthy humans were as follow: one study demonstrated enhanced IL-10 levels (dosage of 3 g/day CLA and observation period of 12 weeks) [32], while another study demonstrated an increase in T lymphocyte proliferation, but not lymphocyte subpopulations (dosage 2.5 g/d and observation period of 8 weeks) [31]. The effects of CLA supplementation on healthy individuals after vaccinations were as follow: healthy men after hepatitis B vaccination (dosage of 3 g/day and observation period of 12 weeks) had improved adaptive (humoral) immune response (an increased seroprotection rate), but other immune functions were not affected [14,46]; healthy women after influenza vaccination (dosage of 3 g/day and observation period of 63 days) had no enhanced immune responses [14,47]. The current trial on HPV-induced LP patients also failed to demonstrate immune enhancement with CLA supplementation after surgery (dosage of 2.5 g/d and observation period of 8 weeks). Discrepancies among previous studies were attributed to different methods, supplementation procedures and subjects used. In the case of the current preliminary study stress (repeated surgical procedures) may have played a role and the small patient number definitely undermined correlations and the statistical power of the trial.

The questions that remain are why do some children have papilloma recurrence and what role has enhanced IL-10 to play in this phenomenon? RLP is considered a complex multigene disease, manifesting as a tissue and HPV-specific immune deficiency, which prevents effective clearance and/or control of HPV6/11 infections [48]. Advanced studies revealed that polymorphisms in the IL-10 gene proximal promotor region are known to influence the production of IL-10 [45]. However, IL-10 is considered: an ant-inflammatory agent; and a critical modulator of the T_H_1/T_H_2 balance that stimulates functions of innate and T_H_2-related (humoral) immunity, but suppresses T_H_1-related (cellular) immune responses [45]. It is therefore conceivable that: enhanced IL-10 can fight viral infections effectively in mildly/moderately aggressive papillomatosis; and that enhanced IL-10 levels during prolonged exposure to viral infections in highly/severely aggressive papillomatosis apparently tilt the scale towards a T_H_2 disease, with consequent immnunosuppression and papilloma recurrence. The link between enhanced IL-10 levels and clearance or failure to clear viral infections in LP patients needs further elucidation.

## Conclusions

Overall, CLA and HOSF treatments contributed to disease outcomes during this one-year crossover study, as witnessed by the reduction in number/no surgical procedures per year in the clinical course of children with LP. After CLA treatment cases with mildly/moderately aggressive papillomatosis were clear of viral infections and they recovered after the one-year trial, depending on viral loading. Cases with highly/severely aggressive papillomatosis had no surgical procedures during treatments, but after the treatment period they had repeated surgical procedures and disease conditions were considered the same. The effects of HOSF were also restricted to mildly/moderately aggressive papillomatosis, but were less effective. Preliminary group findings indicated a significant reduction in the number of surgical procedures per year for cost effective treatment. Disease staging was also improved by significant down-regulation of papillomatosis and amelioration of viral infections after CLA treatment. No significant immune enhancement could be demonstrated after CLA treatment or during the trial. The role of viral loading (rather than HPV type) in disease outcomes requires further clarification. The assumption that there is a link between IL-10 and clearance or failure to clear viral infections after CLA treatment in LP patients needs to be confirmed or refuted.

The preliminary findings of this study remain a valuable contribution to further research for the therapeutic potential of CLA in HPV-induced LP patients. The present study paves the way for large scale trials and prospective development of novel CLA therapy in the treatment regime of HPV-induced LP patients who may qualify as candidates.

## Abbreviations

CLA: Conjugated linoleic acid; HOSF: High oleic safflower oil; Ig: Immunoglobulin; IL: Interleukin; LP: Laryngeal papillomatosis; RLP: Recurrent laryngeal papillomatosis.

## Competing interest

I declare that I have no competing interest.

## Supplementary Material

Additional file 1 **Recurrent laryngeal papillomatosis.** CD counts. Click here for file

Additional file 2 **Recurrent laryngeal papillomatosis.** lmmunoglobulin counts. Click here for file

Additional file 3 **Recurrent laryngeal papillomatosis.** Cytokine counts. Click here for file
